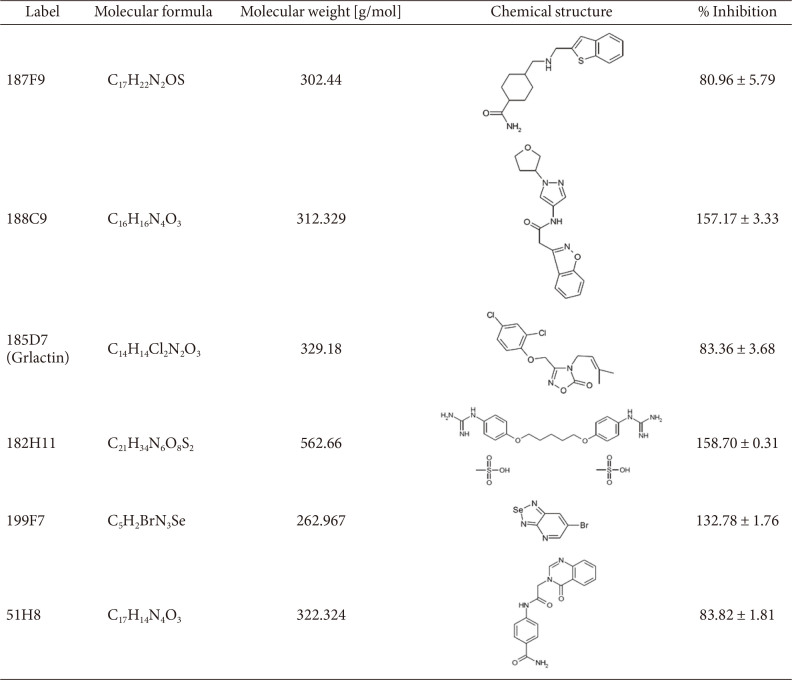# Screening of Anti-Adhesion Agents for Pathogenic *Escherichia coli* O157:H7 by Targeting the GrlA Activator

**DOI:** 10.4014/jmb.2207.07065

**Published:** 2023-01-28

**Authors:** Sin Young Hong, Byoung Sik Kim

**Affiliations:** Department of Food Science and Biotechnology, Ewha Womans University, Seoul 03760, Republic of Korea

**Keywords:** Antibiotic alternatives, *Escherichia coli* O157:H7, EHEC, high-throughput screening (HTS), LEE operon, host cell adhesion

## Abstract

Enterohemorrhagic *Escherichia coli* (EHEC) is a foodborne pathogen that produces attaching and effacing lesions on the large intestine and causes hemorrhagic colitis. It is primarily transmitted through the consumption of contaminated meat or fresh produce. Similar to other bacterial pathogens, antibiotic resistance is of concern for EHEC. Furthermore, since the production of Shiga toxin by this pathogen is enhanced after antibiotic treatment, alternative agents that control EHEC are necessary. This study aimed to discover alternative treatments that target virulence factors and reduce EHEC toxicity. The locus of enterocyte effacement (LEE) is essential for EHEC attachment to host cells and virulence, and most of the LEE genes are positively regulated by the transcriptional regulator, Ler. GrlA protein, a transcriptional activator of *ler*, is thus a potential target for virulence inhibitors of EHEC. To identify the GrlA inhibitors, an in vivo high-throughput screening (HTS) system consisting of a GrlA-expressing plasmid and a reporter plasmid was constructed. Since the reporter luminescence gene was fused to the *ler* promoter, the bioluminescence would decrease if inhibitors affected the GrlA. By screening 8,201 compounds from the Korea Chemical Bank, we identified a novel GrlA inhibitor named Grlactin [3-[(2,4-dichlorophenoxy)methyl]-4-(3-methylbut-2-en-1-yl)-4,5-dihydro-1,2,4-oxadiazol-5-one], which suppresses the expression of LEE genes. Grlactin significantly diminished the adhesion of EHEC strain EDL933 to human epithelial cells without inhibiting bacterial growth. These findings suggest that the developed screening system was effective at identifying GrlA inhibitors, and Grlactin has potential for use as a novel anti-adhesion agent for EHEC while reducing the incidence of resistance.

## Introduction

Although antibiotics have been effective at controlling diverse bacterial pathogens, the selective pressure on bacterial growth resulted in the emergence and spread of antibiotic-resistant pathogens [1-3]. According to the “Jim O'Neill Report” released by the British government in 2016, approximately 10 million people worldwide die every year due to infection with antibiotic resistant bacteria [4]. However, simply lowering the use of antibiotics would not solve the issue, and complete prevention of antibiotic use is not possible. Therefore, it is critical to develop a new pathogen-controlling strategy that does not induce resistance. Accordingly, toxin function/delivery, bacterial attachment, and virulence mechanisms have been studied as control targets for disrupting host-pathogen interactions [1].

Enterohemorrhagic *Escherichia coli* (EHEC) is a foodborne pathogen that can cause bloody diarrhea and hemorrhagic colitis. In severe cases, the infection may progress to hemolytic uremic syndrome (HUS) [5-7]. The primary virulence factor responsible for this acute renal failure is a phage-encoded Shiga toxin (alternatively referred to as Verotoxin), although pathological mechanism of the intoxication is barely disclosed, yet [8]. Consistent with the fact that bovine intestinal tract is the principal environmental reservoir for this pathogen, many EHEC outbreaks are associated with raw or undercooked meat and unpasteurized milk. Fresh leafy greens, such as romaine lettuce and radish sprouts, are also responsible for the outbreaks [9]. Since the outbreaks occur frequently in summer, strict management is required during this season. However, antibiotic treatment for EHEC infections is contraindicated because it may result in a severe HUS case through induction of the Shiga toxin-encoding prophages [10]. Similar to other pathogens, the spread of antibiotic resistance is also of great concern for EHEC. Consequently, additional efforts on developing antibiotic alternatives are urgently required to control EHEC.

**Fig. 1:**
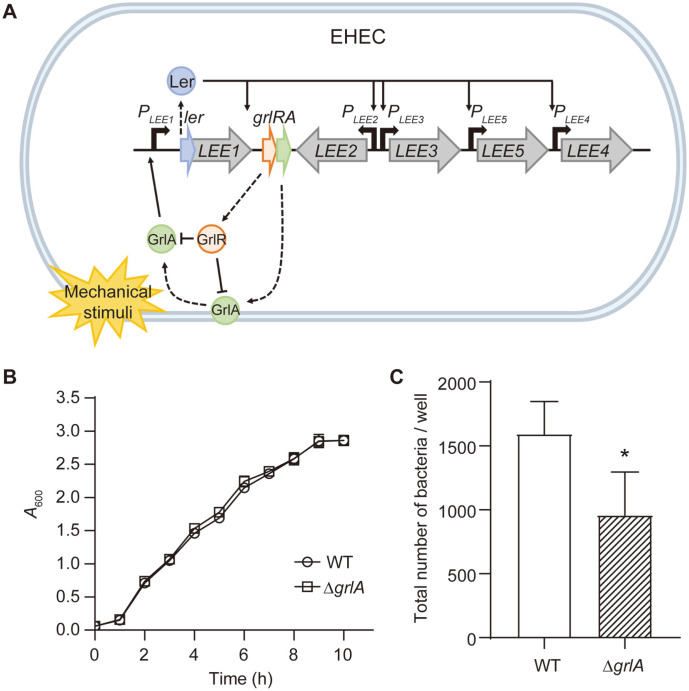
Validation of GrlA as a target for an anti-adhesion agent. (**A**) Schematic diagram of the GrlA-mediated regulation of LEE operons. The LEE operons (*LEE1* to *LEE5*) and a bicistronic *grlRA* operon located between *LEE1* and *LEE2* are shown. When EHEC detects mechanical stimuli in the host intestine during initial attachment, membrane-bound inactive GrlA is released from the membrane and moves to the cytoplasm where it can activate the transcription of *ler* by binding to the *LEE1* promoter. The expressed Ler then activates LEE operons as well as *grlRA*. GrlR interacts with the helix-turn-helix motif of GrlA and inhibits it. (**B**) Growth curve of *E. coli* EDL933 WT and Δ*grlA*. The strains were cultured in LB medium at 37°C, and the *A*_600_ was measured hourly. (**C**) HeLa cells were cultured on a 24-well cell culture plate and co-incubated with the indicated EDL933 strains at an MOI of 100. After 2 h, the bacteria bound to the HeLa cells were quantified and expressed as the number of bacteria per well. The data were represented as the means ± standard deviation (SD) from three independent experiments. The Student’s t-test was used to determine the statistical significance (*, *p* < 0.05).

EHEC colonizes the large intestine and forms attaching and effacing (A/E) lesions by destroying microvilli and rearranging the actin cytoskeletons of enterocytes to form pedestal-like structures, to which the pathogen adheres [11]. In addition to EHEC, enteropathogenic *E. coli* (EPEC) and *Citrobacter rodentium* also form A/E lesions and, thus, belong to the A/E pathogens [12]. Notably, these pathogens possess the locus of enterocyte effacement (LEE) pathogenicity island, where genes associated with the A/E lesion are encoded [13-15]. The LEE consists of more than 40 open reading frames (ORFs) that are functionally organized into five major operons from *LEE1* to *LEE5* [16] (Fig. 1A). *LEE1*, *LEE2*, and *LEE3* harbor the *esc* and *sep* genes, which encode the main structural proteins of the type 3 secretion system (T3SS) [17]. The genes in the *LEE4* operon encode several T3SS effector proteins [18]. Finally, the *LEE5* operon contains the *eae* and *tir* genes, which encode intimin and Tir, respectively. Similar to other effector proteins, Tir is also delivered into host cells through T3SS and contributes close adhesion of EHEC to the intestinal cells by directly interacting with intimin molecules expressed at the pathogen surface. To coordinately regulate the expression of all these LEE operons, the LEE encoded regulator (Ler) in the *LEE1* operon, functions as a major transcriptional regulator of the LEE operons [19].

The *grlRA* genes, located between the *LEE1* and *LEE2* operons, encode a global regulator of LEE activator (GrlA) and a global regulator of LEE repressor (GrlR) [20]. GrlA positively regulates the *ler* gene expression by directly binding to the *LEE1* promoter and functions as an anti-repressor against the H-NS proteins. Furthermore, Ler activates the transcription of *grlRA*, forming a positive feedback loop [21]. For the precise control of this loop, however, the co-expressed GrlR protein sequesters GrlA, preventing unbalanced feedback activation of *LEE1* [20]. Recently, an additional level of regulation for GrlA activity has been discovered. In detail, the initial adherence of EHEC to the host cell surface generates mechanical cues, which induce the expression of LEE genes in a GrlA-dependent manner [22-24]. In response to the mechanical stimulation, a membrane-bound, inactivated form of GrlA migrates to the cytoplasm and binds to the *LEE1* promoter to activate the operon (Fig. 1A) [25]. These GrlA-mediated comprehensive and precise regulations on the LEE pathogenicity island render GrlA an attractive target for an anti-virulence agent.

To date, several inhibitor molecules for the transcriptional regulator Ler, such as yomogin and diosmin, have been identified [26, 27]. However, no compound has been discovered that targets and controls the GrlA activator. Consequently, we constructed reporter strains to detect the GrlA inhibitors and screened a total of 8,201 compounds. The screened-out inhibitor, Grlactin, was validated for its activity by applying it to EHEC strain EDL933. The expression level of the representative LEE genes, as well as the number of adherent bacteria on the host cells, were significantly decreased, suggesting that Grlactin has potential as an antibiotic alternative in controlling EHEC.

## Materials and Methods

### Bacterial Strains, Plasmids, Culture Conditions, and Chemicals

The bacterial strains and plasmids used in this study are listed in Table 1. *E. coli* EDL933 and its *grlA* deletion mutant strain (Δ*grlA*) were obtained from Dr. S.H. Choi at Seoul National University. Unless noted otherwise, the *E. coli* strains were grown in a Luria-Bertani (LB) medium at 37°C with the appropriate antibiotics at the following concentrations: Ampicillin, 100 μg/ml; Kanamycin, 100 μg/ml; Chloramphenicol, 20 μg/ml; and Streptomycin, 50 μg/ml. Before the cell adherence assay was conducted, the *E. coli* strains were grown in Dulbecco’s minimal Eagle medium (DMEM; Thermo Fisher Scientific, USA). A total of 8,201 compounds consisting of synthetic and natural molecules, dissolved in dimethyl sulfoxide (DMSO), were obtained from the Korea Chemical Bank (http:/ /www.chembank.org).

### Generation of the grlA* Mutant Strain

The primers used in this study are listed in Table 2. It is known that basic residues on the C-terminus of the GrlA protein (Lys 111 and Arg 114) are involved in mechano-sensing and, thus, are critical for the GrlA-mediated activation of *LEE1* upon mechanical stimulation [25]. To examine whether the hit compounds from the initial screening exhibited GrlA-inhibitory activity regardless of the mechano-sensing status of GrlA, we constructed the mechano-sensing defective mutant grlA*. Briefly, point mutations on the basic residues of *grlA* (K111A, R114A) were introduced into the plasmid-cloned wild-type (WT) *grlA* gene by site-directed mutagenesis, and then the truncated *grlA* locus in the Δ*grlA* mutant strain was substituted with the grlA* using the CRISPR/Cas9 system, as described below.

First, a custom single guide RNA (sgRNA)-expressing plasmid, pTargetF_*grlA*, was constructed (Table 1). For this purpose, near the 5' end of the truncated *grlA* gene sequence in Δ*grlA* was inspected for the N20 region containing a PAM site (nGG) with high %GC content. NEBaseChanger (New England Biolabs, USA; https://nebasechanger.neb.com/) was then used to design the primers required for the introduction of the detected N20 region as a sgRNA sequence into the pTargetF plasmid.

Next, a repair template consisting of the gene to be inserted (grlA*) and 300 bp up- and down-stream homology arms were prepared. The WT *grlA* gene and the flanking regions were cloned into the pBluescript SK II (+) plasmid, and the point mutations (K111A and R114A) were introduced via site-directed mutagenesis. Due to the presence of the sgRNA-matching sequence in the resulting construct, other silence mutations (ATC to ATT for Ile 130 and AGG to CGG for Arg 132) were additionally introduced to the grlA* gene. This manipulation prevents the sgRNA-guided Cas9 protein from recognizing and cutting the matching part after knock-in of the grlA*. The resulting mutated sequence (*grlA* with K111A, R114A, and the silence mutations at Ile 130 and Arg 132) was amplified with the flanking homology arms by PCR and used as a repair template.

Finally, the repair template and pTargetF_*grlA* were co-electroporated into the EDL933 Δ*grlA* strain, which expresses Cas9 from the pCas plasmid. After recovery, the cells were spread on LB agar plates containing kanamycin and streptomycin and incubated overnight at 30°C. The correctly mutated construct was then identified by PCR and sequencing, and the remaining plasmids (pTargetF_*grlA* and pCas) were removed as described previously [28].

### High-throughput Screening (HTS) of Small Molecules

To construct the HTS system, reporter strains carrying the GrlA-expressing plasmid and reporter plasmid were created. The *grlA*, grlA*, and promoter region of *ler* (384 bp) were amplified by PCR and then treated with restriction enzymes. The resulting products and linearized plasmids (pJK1113 for *grlA* and grlA*; pBBR_lux for the promoter region of *ler*) were ligated to create an arabinose-inducible GrlA-expressing plasmid (pSY2101), an arabinose-inducible GrlA*-expressing plasmid (pSY2201), and a reporter plasmid (pSY2104) containing the GrlA-activated *ler* promoter fused to the bioluminescence (*lux*) operon. The heterologous host reporter strains, SY2102 and SY2201, were constructed by co-transforming *E. coli* DH5α with the reporter plasmid pSY2104 and either pSY2101 or pSY2201, respectively. The homologous host reporter strains, SY2110 and SY2204, were constructed by transforming *E. coli* EDL933 having either the WT *grlA* or grlA*, respectively, with the reporter plasmid pSY2104. As a positive control for the homologous host reporter system, SY2111 was constructed by transforming the EDL933 Δ*grlA* strain with the reporter plasmid pSY2104.

The HTS was performed as described previously with a slight modification [29]. The heterologous host reporter strains SY2102 or SY2201 were cultured to an absorbance at 600 nm (*A*_600_) of 0.2 in fresh LB containing 0.0001% or 0.0002% L-(+) arabinose, respectively, with the appropriate antibiotics. One hundred microliters of culture were transferred to each well of a 96-well microtiter plate containing a 20 μM concentration of each molecule or 2% DMSO. The plates were incubated at 37°C with shaking, and the relative luminescence unit (RLU) was measured three times at hourly intervals using a Spark microplate reader (Tecan, Switzerland). For these screenings, the DMSO-treated reporter *E. coli* without L-(+) arabinose was set as a positive control, while the DMSO-treated reporter *E. coli* with L-(+) arabinose was set as a negative control. The homologous host reporter strains and their positive control strain SY2111 were cultured to an *A*_600_ of 0.2 (for SY2110 and SY2111) or 0.5 (for SY2204 and SY2111) in fresh LB with the appropriate antibiotics, and the RLU value was calculated hourly. The percentage of inhibition was calculated using the following equation:

$$\text{\% Inhibition = 100×}\frac{\text{(RLU of the sample treated with a compound-RLU of the negative control)}}{\text{(RLU of the positive control-RLU of the negative control)}}$$

### Cell Culture and Bacterial Adherence Assay

HeLa cells (ATCC CCL-2) were kindly provided by Dr. S.H. Choi at Seoul National University and cultured at 37°C with 5% CO_2_ in DMEM medium supplemented with 10% heat-inactivated fetal bovine serum (FBS; Thermo Fisher Scientific) and 1% antibiotic/antimycotic (Thermo Fisher Scientific). Cell adhesion assays were performed using the previously described method [30], with a slight modification. Briefly, the HeLa cells were seeded and grown in 24-well cell culture plates until confluent. Before bacterial treatment, monolayers of the HeLa cells were washed three times with pre-warmed Dulbeccós Phosphate Buffered Saline (DPBS, Thermo Fisher Scientific), and the fresh DMEM without antibiotic/antimycotics and FBS were added to each well. The HeLa cells were then treated with the indicated bacterial strains at a multiplicity of infection (MOI) of 100 (2 × 10^7^ CFUs/well) and further incubated for 2 h in the presence (20 μM) or absence of hit molecules at 37°C with 5% CO_2_. After washing out the non-adherent bacterial cells with DPBS, the HeLa cells were lysed with 1% Triton X-100. Finally, serial dilutions of the lysates were plated on LB agar and incubated overnight at 37°C. The colonies were counted the next day to determine the total number of adherent bacteria per well.

### Total RNA Extraction and Quantitative Real-Time PCR (qRT-PCR)

The preparation process for total RNA extraction from the HeLa cell-attached EDL933 was described elsewhere [31]. Briefly, the HeLa cells were seeded in a 24-well cell culture plate at a density of 2 × 10^5^ cells per well and treated with EDL933 at an MOI of 100, as described above. After 2 h of co-incubation in the presence or absence of hit molecules, the HeLa cells were washed three times with DPBS to remove the non-adherent bacteria. To collect the total RNA from the attached bacteria, 400 μl of DPBS and 800 μl of RNAProtect Bacteria Reagent (Qiagen, Germany) were added to each well, followed by scraping and centrifugation of the cells. The pellet was kept at -20°C until total RNA extraction. The total RNA was extracted from the pellets using an RNeasy kit (Qiagen), and all the samples were treated with DNaseI to remove any contaminated genomic DNA. The extracted RNA was kept at -80°C. The quantity and quality of the RNA in the samples were determined using a NanoQuant plate and Spark microplate reader (Tecan).

For qRT-PCR analysis, complementary DNA (cDNA) was synthesized using the iScript Reverse Transcription Supermix (Bio-Rad Laboratories, USA). For each well, 10 μl of 2X iQ SYBR supermix (Bio-Rad), 0.5 μl of forward primer, 0.5 μl of reverse primer, 7 μl of Nuclease-free water, and 2 μl of cDNA template were added, and amplification was performed using QuantStudio Real-Time PCR System (Applied Biosystems, USA). The genes that were closest to the promoter of each LEE operon were examined. The relative expression levels of the transcripts were calculated using the expression level of the *rpoA* housekeeping gene as the internal reference for normalization. The specific primers used for the amplification of the cDNAs are listed in Table 3.

### Statistical Analysis

The data from at least three independent experiments were expressed as the mean ± standard deviation (SD). Statistical analyses were performed using GraphPad Prism software version 7.0. The Student’s *t*-test or one-way ANOVA with multiple comparison was used to determine the statistical significance among experimental groups, as noted in each figure legend.

## Results

### Validation of GrlA as a Target for the EHEC Adherence Inhibitor

When grown in LB medium, the *E. coli* EDL933 Δ*grlA* strain did not show any growth defects (Fig. 1B). This suggests that even if a potential inhibitor molecule affected the GrlA protein, the possibility for selective pressure and emergence of resistance is low. Next, the efficacy of the GrlA inhibition on the EHEC virulence was estimated by comparing the WT and Δ*grlA* strains for their host cell-adhering activity. After 2 h of co-incubation of the bacterial cells and HeLa cells, the amount of the HeLa cell-adherent Δ*grlA* strain was 1.7-fold lower than that of the WT *E. coli* EDL933 (Fig. 1C). This indicates that the reduced adherence of EDL933 on the host cells could be achieved via GrlA inhibition, most likely by lowering the expression of LEE genes. Overall, GrlA was validated as an attractive target for an EHEC adherence inhibitor.

### Construction of the Reporter Strain for HTS

**Fig. 2:**
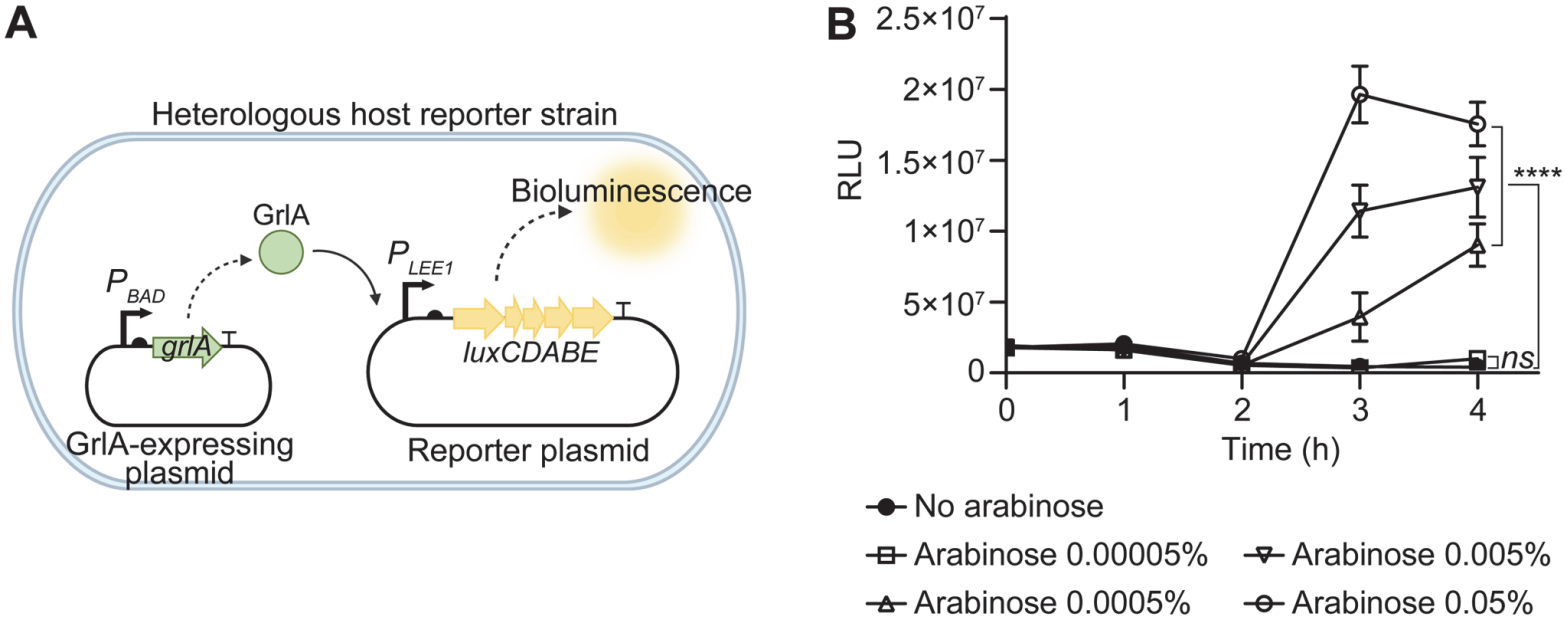
Reporter strain for the HTS of the GrlA inhibitor. (**A**) A heterologous host *E. coli* DH5α reporter strain contains pSY2101 expressing GrlA under the P_BAD_ promoter and pSY2104 carrying *luxCDABE* genes under the GrlA-activated promoter *P_LEE1_*. (**B**) The constructed reporter strain was verified by L-(+) arabinose induction, with various concentrations (w/ v) indicated. The one-way ANOVA with multiple comparison was used to determine the statistical significance between the control (no arabinose) and other samples at 4h (ns, non-significant; ****, *p* < 0.0001).

To screen the chemical libraries for GrlA inhibitors, an HTS system was constructed, as described in the Materials and Methods section. Since the *E. coli* DH5α strain lacks the Ler, GrlA, and LEE operons, this heterologous host would enable us to investigate the GrlA-dependent response of LEE gene expression with few potential crosstalks or signal inputs mediated by other regulatory proteins and pathways. Therefore, the heterologous host (*E. coli* DH5α) reporter strain was constructed by co-transforming an arabinose-inducible GrlA-expressing plasmid (pSY2101) and a GrlA-dependent *lux* reporter plasmid (pSY2104) (Fig. 2A). Since GrlA binds directly to the *LEE1* promoter to activate the transcription of *ler*, the expression of the *lux* operon would be diminished if potential hit molecules interfered with the activity of GrlA. The feasibility of the construct as a reporter strain was examined via treatment with various concentrations of arabinose, and varying levels of bioluminescence emission were confirmed depending on the arabinose concentration (Fig. 2B).

### HTS Screening Using WT *grlA* Reporter Strains

**Fig. 3:**
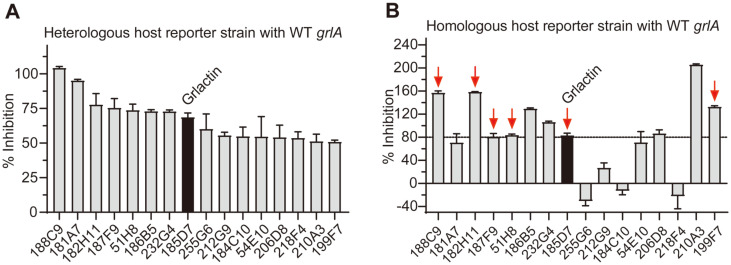
Validation of the initial hit molecules from the HTS. (**A**) Validation using the heterologous host reporter strain SY2102. The strain was grown in LB medium supplemented with 0.0001% L-(+) arabinose and 2% DMSO or each chemical (final concentration of 20 μM). As a positive control, SY2102 was grown without L-(+) arabinose. (**B**) Validation using the homologous host reporter strain SY2110. The strain was grown in LB medium supplemented with 2% DMSO or each chemical (20 μM). As a positive control, SY2111 was grown in LB medium supplemented with 2% DMSO. After 5 h of incubation, the luminescence and absorbance were measured to calculate the % inhibition. The data were represented as the means ± SD from three independent experiments. The red arrows indicate the selected six compounds at this validation step with the homologous host reporter strain SY2110.

To identify the anti-virulence molecule that prevents EHEC adherence to host cells via GrlA inhibition, a total of 8,201 compounds from the Korea Chemical Bank were screened at a concentration of 20 μM using the heterologous host reporter strain SY2102. After the initial screening, 80 compounds with a 50 or higher %inhibition were classified as “possible hits” and examined further in triplicate. It should be noted that the compounds that showed reproducible results in the triplicate experiments were selected as “initial hits.” The compounds that showed growth inhibition were excluded from the hits because the purpose of this HTS was to identify the molecules that would not result in selective pressure on *E. coli*. Colored chemicals were also excluded because the read-out of the screening system was based on bioluminescence. Consequently, a total of 16 compounds were selected as initial hit molecules (Fig. 3A).

The molecules were further validated using the homologous host reporter strain SY2110, and 6 of 16 compounds were chosen at this step (Fig. 3B). The selection criteria were: compounds that exhibited greater than 80% inhibition and were not toxic to the homologous host reporter strain. The molecular characteristics, such as the chemical structure and molecular weight, of these six compounds are listed in Table 4.

### Validation Using grlA* Reporter Strains

**Fig. 4:**
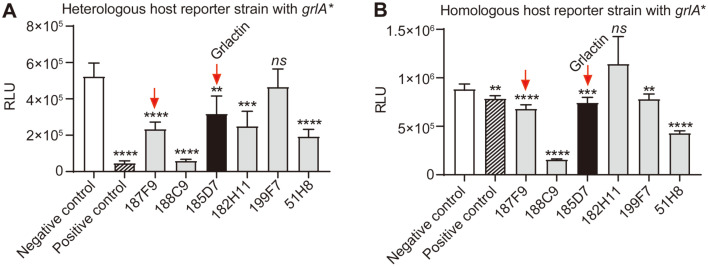
Validation of the hit molecules using strains expressing grlA*. An additional set of heterologous (SY2201) and homologous (SY2204) host reporter strains expressing grlA* were prepared and used for the hit validation. (**A**) Strain SY2201 was grown in LB medium supplemented with 0.0002% L-(+) arabinose and the indicated hit molecules (20 μM). The negative and positive controls were the same strain grown with or without L-(+) arabinose, respectively. (**B**) Strain SY2204 was grown in LB with the indicated hit molecules (20 μM). Strains SY2204 and SY2111 treated with 2% DMSO were used as a negative and positive control, respectively. The RLU of each sample was calculated after 4 h of incubation. The data were represented as the means ± SD from three independent experiments. The red arrows indicate the selected two compounds at these validation steps. The Student’s *t*-test was used to determine the statistical significance between the negative control and other samples (ns, non-significant; ****, *p* < 0.0001; ***, *p* < 0.001; **, *p* < 0.01).

GrlA is a membrane-bound, mechano-sensing transcriptional regulator, the mechanism of which is liberation from the bacterial inner membrane [25]. Therefore, a possible scenario for the selected hits is that the inhibitor traps the GrlA in the inner membrane. To examine this scenario, we introduced point mutations (K111A and R114A) in the *grlA* that resulted in the expression of a membrane binding-defective mutant, GrlA*. All six compounds were then evaluated using the heterologous and homologous host reporter strains harboring grlA*. As shown in Figs. 4A and 4B, the compounds 199F7 and 182H11 did not exhibit inhibition activity either for the heterologous or homologous host reporter strains with grlA*, respectively, suggesting that they may affect the liberation of GrlA from the membrane, not the binding of GrlA to the *LEE1* promoter. The compounds 188C9 and 51H8 were also excluded from the further investigation since both excessively reduced the RLU beyond the level of the positive control, suggesting an adverse effect on the reporter plasmid maintenance/replication and not GrlA (Fig. 4B). From the screening and validation, two hit molecules (187F9 and 185D7) were selected as putative GrlA inhibitors.

### Grlactin Diminishes EHEC Adhesion on Host Cells by Reducing the Expression of LEE Genes

**Fig. 5:**
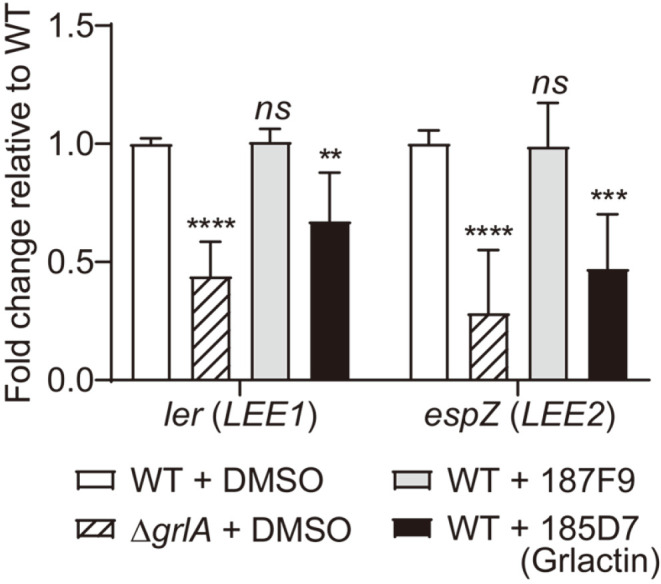
GrlA inhibitor, Grlactin. The relative expression levels of the *ler* (*LEE1*) and *espZ* (*LEE2*) genes from the indicated samples were determined by qRT-PCR and expressed using that from the DMSO-treated WT strain sample as 1. The data were represented as the means ± SD from three independent experiments. The Student’s *t*-test was used to determine the statistical significance between the DMSO-treated WT and other samples (ns, non-significant; ****, *p* < 0.0001; ***, *p* < 0.001; **, *p* < 0.01).

All the above results were obtained from artificial reporter systems. Therefore, two compounds were examined again to assess whether they reduced the expression of the *ler* gene and the Ler-regulated *espZ* gene, which is encoded in the *LEE2* operon. When *E. coli* EDL933 was treated with 187F9 or 185D7 for 2 h in the presence of HeLa cells, only 185D7 reduced the expression of both *ler* and *espZ* compared to the levels in the Δ*grlA* control strain (Fig. 5). Based on this result, the compound 185D7 [3-[(2,4-dichlorophenoxy)methyl]-4-(3-methylbut-2-en-1-il)-4,5-dihydro-1,2,4-oxadiazole-5-on] was selected as a GrlA inhibitor and was renamed Grlactin.

**Fig. 6:**
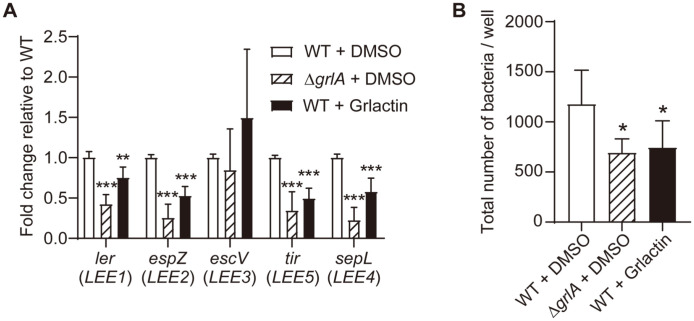
Effects of Grlactin on the expression of the LEE genes and the adhesion of EHEC to the host cells. (**A**) Effects of Grlactin on the expression level of each LEE operon gene were examined by qRT-PCR. (**B**) Reduction in EHEC adhesion to the HeLa cells after Grlactin treatment. The data were represented as the means ± SD from three independent experiments. The Student’s *t*-test was used to determine the statistical significance between the DMSO-treated WT and other samples (***, *p* < 0.001; **, *p* < 0.01; *, *p* < 0.05).

Next, the effects of Grlactin on the expression of the LEE operons were examined. For each operon, the *ler* (*LEE1*), *espZ* (*LEE2*), *escV* (*LEE3*), *tir* (*LEE5*), and *sepL* (*LEE4*) genes located very downstream of each LEE promoter were selected, and their expression in the presence of Grlactin was compared with the DMSO-treated control strains. Grlactin significantly downregulated the expression of *ler*, *espZ*, *tir*, and *sepL* by 1.3-fold to 2.0-fold (Fig. 6A). However, the expression of *escV*, which is located on *LEE3*, was highly inconsistent in the presence of Grlactin, which was similar to the DMSO-treated Δ*grlA* control (Fig. 6A).

One of the known LEE operon-mediated pathological phenotypes in EHEC is host cell adhesion [19]. Consistent with this, Grlactin significantly diminished EHEC adhesion on HeLa cells, which resembled that of the DMSO-treated Δ*grlA* control. Indeed, after genetic knock-out of *grlA* or Grlactin treatment, the number of EHEC cells adhered to the HeLa cells was reduced by 1.7-fold and 1.6-fold, respectively (Fig. 6B).

## Discussion

Based on the molecular version of Koch’s postulation, an anti-virulence strategy has been proposed and explored for various virulence factors in diverse pathogens [1, 29, 32-34]. Here, we constructed an HTS system to detect substances that inhibit the EHEC adherence on host cells. Hence, a transcriptional activator GrlA, which comprises a crucial positive feedback loop with Ler for LEE expression, was targeted. Since GrlA is activated when it senses mechanical stimuli in the intestinal environment of a host (such as fluid shear), and because GrlA-mediated LEE expression (particularly *LEE5*-encoded intimin and Tir expression) is essential for tight adhesion of EHEC on intestinal epithelial cells, the inhibition of GrlA could prevent the extremely early stage of EHEC infection in the host intestine [25].

Although the crystal structure of the full-length GrlA has not yet been solved, the structure of a truncated GrlA, with its carboxyl 31 residues omitted (GrlAΔ, 1-106 amino acids), is available in the form of the GrlR-GrlAΔ complex [35]. In such a structure, no ligand molecule(s) or distinct ligand-binding pockets were detected. Accordingly, we screened a random chemical library rather than a focused library using the constructed HTS system to discover GrlA inhibitors.

The selected inhibitor, Grlactin, significantly downregulated the expression levels of the examined LEE genes (except for *escV*) when the bacteria were co-incubated with HeLa cells (Fig. 6A). A previous study revealed that the promoter regions, specifically the -10 boxes, of the divergently transcribed *LEE2* and *LEE3* operons are overlapped [36]. In general, this region is covered by the histone-like nucleoid structuring protein H-NS and, thus, the expression of *LEE2* and *LEE3* is principally repressed. After the environmental signal from the host, however, the Ler protein is induced and directly binds to the overlapping promoter region, releveling the H-NS-mediated negative regulation [37]. In this scenario, the expression of both *LEE2* and *LEE3* should be diminished if the Grlactin affected the GrlA activity for the *ler* induction. Then, why does the *escV* encoded in the *LEE3* operon exhibit such a unique response? One possible explanation is that GrlA may function at this regulatory region to activate the transcription of *LEE2* but not *LEE3* in a Ler-independent manner. Consistent with this, a previous study demonstrated that the transcription level of *LEE2* increased and that of *LEE3* decreased when both Ler and GrlA were present at the same time [38]. Although further study is required to elucidate the exact role of GrlA in *LEE3* transcription, the results of the present study showed that the inhibitor Grlactin can have an adverse effect on the precise control of various virulence genes in the LEE pathogenicity island (Fig. 6A).

Lastly, the consequence of Grlactin treatment on EHEC adhesion was examined using HeLa cells. As expected, the number of bacteria attached to the HeLa cells was significantly reduced (by approximately 1.6-fold) after treatment with Grlactin (20 μM; Fig. 6B). However, it should be noted that the degree of reduction was less than that observed in previous studies, in which yomogin [26] or diosmin [27] was used as a treatment. When Caco-2 epithelial cells were co-incubated with EHEC, the Ler-targeting yomogin (0.05 μg/μl; approximately 204.67 μM) or diosmin (100 μM) treatment decreased the bacterial adhesion by approximately 2.7-fold or 3.6-fold, respectively. In addition to the difference in the inhibitor concentrations, the distinct target for each inhibitor may be a contributing factor to the difference in the efficacy. In fact, GrlA indirectly regulates the LEE operons through Ler, while Ler directly regulates the operons. Furthermore, many regulators, other than GrlA, activate or repress the transcription of *ler* by acting on the *LEE1* promoter [39]. Nonetheless, Grlactin remains a promising agent to target EHEC virulence, particularly considering the possible synergistic effects with yomogin or diosmin. Since Grlactin inhibits a distinct protein GrlA, which forms a positive feedback loop with Ler for LEE gene expression, it can be used together with such Ler inhibitors to potentiate their anti-virulence activity. Of note, Grlactin does not inhibit bacterial growth like yomogin or diosmin. Future studies are required to examine the possible synergism among the molecules.

Overall, this study demonstrates the small molecule Grlactin as an antibiotic alternative that downregulates LEE gene expression and the initial adhesion of EHEC onto host cells by affecting GrlA function. Grlactin is a synthetic compound, and information on this molecule is limited. It has the potential to be developed as a novel anti-adhesion agent for EHEC; however, future research should include a safety assessment and mechanistic studies, such as the effects of Grlactin on the binding of GrlA to its target promoter DNA.

## Figures and Tables

**Table 1:** Bacterial strains and plasmids used in this study.

Strain or plasmid	Relevant characteristics^a^	Reference or source
Bacterial strain		
*E. coli*		
EDL933 (ATCC 43895)	Wild type, clinical isolate, virulent	Laboratory collection
EDL933 Δ*grlA*	EDL933 with *grlA* deletion mutation	Seoul Nat’l Univ.
EDL933 grlA*	EDL933 with K111A, R114A mutations introduced into chromosomal *grlA*	This study
DH5α	*E. coli* K-12 strain for cloning experiments	Laboratory collection
SY2102	DH5α containing pSY2101 and pSY2104; Ap^r^, Km^r^, Cm^r^	This study
SY2110	EDL933 containing pSY2104; Cm^r^	This study
SY2111	EDL933 Δ*grlA* containing pSY2104; Cm^r^	This study
SY2201	DH5α containing pSY2201 and pSY2104; Ap^r^, Km^r^, Cm^r^	This study
SY2204	EDL933 grlA* containing pSY2104; Cm^r^	This study
Plasmids		
pJK1113	pBAD24 with oriT of RP4 and nptI, P_BAD_; Ap^r^, Km^r^	[40]
pBBR_lux	Broad host range vector with promoterless *luxCDABE*; Cm^r^	[41]
pSY2101	pJK1113 with *grlA*; Ap^r^, Km^r^	This study
pSY2104	pBBR_lux with a fragment of *LEE1*/*ler* upstream region; Cm^r^	This study
pSY2201	pJK1113 with grlA*; Ap^r^, Km^r^	This study
pBluescript SK II (+)	pUC ori, *lacZ*; Ap^r^	Agilent
pCas	*repA101*(Ts) *kan* *P_cas_*-*cas9* *P_araB_*-*Red* *lacI*^q^ *P_trc_*-sgRNA-*pMB1*	[42]
pTargetF	*pMB1* sgRNA_*pMB1*; Str^r^	[42]
pTargetF_*grlA*	*pMB1* sgRNA_*grlA*; Str^r^	This study

^a^Ap^r^, ampicillin resistant; Km^r^, kanamycin resistant; Cm^r^, chloramphenicol resistant; Str^r^, streptomycin resistant.

**Table 2:** Oligonucleotides used in this study.

Oligonucleotide^a^	Oligonucleotide sequence, 5’-3’	Use(s)
GrlA_NcoI_F	TCAGCCATGGAATCTAAAAATAAAAATGGCG	Expression plasmid construction
GrlA_XbaI_R	AGCTCTAGACTAACTCTCCTTTTTCCGC	Expression plasmid construction
P*ler*_SacI_F	CATCGAGCTCTATAGTGAAACGGTTCAGCTTGG	Reporter plasmid construction
P*ler*_BamHI_R	GCGGGATCCAATAAATAATCTCCGCATG	Reporter plasmid construction
*grlA*_ups_HindIII_F	CCCAAGCTTCTGCTATAGTGAAGTGCTC	Repair template insertion
*grlA*_downs_XbaI_R	GCTCTAGATATTGCTTCTGTGTATCAGGG	Repair template insertion
*grlA*_pTarget_F	GATCATGAGGGTTTTAGAGCTAGAAATAGC	pTarget sgRNA Cloning
*grlA*_pTarget_R	ATTTCGTTCCACTAGTATTATACCTAGGAC	pTarget sgRNA Cloning
*grlA*_ML1_SDM_F	CCGGACCAAAAAGAGCAACCTACGCGGTTGGTAATGGTATTG	Site-directed mutagenesis (ML1)
*grlA*_ML1_SDM_R	CAATACCATTACCAACCGCGTAGGTTGCTCTTTTTGGTCCGG	Site-directed mutagenesis (ML1)
*grlA*_X1_F	AAGATGAGGACATCATACTTCATG	Repair template
*grlA*_X1_R	ACCGACCACTGGTAGATTTATC	Repair template
*grlA*_N20_SDM_F	GGAACGAAATGATTATGCGGCGGAAAAAGGAG	Site-directed mutagenesis (N20)
*grlA*_N20_SDM_R	CTCCTTTTTCCGCCGCATAATCATTTCGTTCC	Site-directed mutagenesis (N20)
grlA*_conf_F	GAAGGTATACTGATTAAAAATGG	grlA* confirmation
grlA*_conf_R	CCATCAGCTATACCAATCC	grlA* confirmation

^a^The oligonucleotides were designed using the genome sequence of *E. coli* O157:H7 str. EDL933. (GenBank accession numbers NZ_CP008957.1).

**Table 3:** Oligonucleotides used for quantitative real-time PCR.

Oligonucleotide	Oligonucleotide sequence, 5’-3’^a^	

	Forward	Reverse
*rpoA* Housekeeping gene	GTGGAGCGTATTGCCTACAA	GTGCCGTTGGTTTCCATTTC
*ler*	CGAGAGCAGGAAGTTCAAAGT	AGTCCATCATCAGGCACATTAG
*espZ*	CGACCTCACTCAGTGGAAATAA	GGTAAGTGCTAATCCGGCTATAA
*escV*	GTCTTCTTATTTCTGGCGGTTTG	CAGCTCCCATAGCATCCTTATT
*tir*	CGCACGTACGGTAGAGAATAAG	GTCTGCTCTCCATGGTATCTTC
*sepL*	CGGGTATCGATTGTCGAAGATAA	GCATTCTCTCTCTGCTCACTATC

^a^The oligonucleotides were designed using the *E. coli* O157:H7 str. EDL933 genome sequence (GenBank accession numbers NZ_CP008957.1).

**Table 4:** Molecular characteristics of the hit compounds.